# Boi-ogi-to (TJ-20), a Kampo Formula, Suppresses the Inflammatory Bone Destruction and the Expression of Cytokines in the Synovia of Ankle Joints of Adjuvant Arthritic Rats

**DOI:** 10.1155/2017/3679295

**Published:** 2017-05-07

**Authors:** Xinwen Zhang, Zhou Wu, Yicong Liu, Junjun Ni, Chunfu Deng, Baohong Zhao, Hiroshi Nakanishi, Jing He, Xu Yan

**Affiliations:** ^1^Center of Implant Dentistry, School of Stomatology, China Medical University, Shenyang 110002, China; ^2^Department of Aging Science and Pharmacology, Faculty of Dental Science, Kyushu University, Fukuoka 812-8582, Japan; ^3^The VIP Department, School of Stomatology, China Medical University, Shenyang 110002, China

## Abstract

TJ-20 is a formula consisting of 6 herbs that has been used in the clinical treatment of rheumatoid arthritis (RA) in China and Japan for centuries. However, scientific evidence of the effects of TJ-20 has not been established. In the present study, we focused on the therapeutic effects and investigated the main function of TJ-20 on adjuvant arthritis (AA), an animal model of RA, which was induced with complete Freund's adjuvant (CFA). TJ-20 was administered orally at 600 mg/kg once a day from 0, 7, and 10 days to 8 weeks after the CFA treatment. TJ-20 significantly ameliorated inflammatory progression and bone destruction in AA in a time-dependent manner. Furthermore, TJ-20 significantly reduced the increased changes in a number of macrophages and helper T cells. Moreover, TJ-20 suppressed the expression of TNF-*α* whereas it augmented the expression of IL-10 and attenuated Th1 cells responses in the synovia of the ankle joint. Therefore, TJ-20 regulated the expression of proinflammatory and anti-inflammatory cytokines in macrophages and Th1/Th2 balance in the synovia of ankle joints in AA rats. These results suggest the positive anti-inflammatory effect of TJ-20 and provide a scientific basis for the clinical use of TJ-20 for RA.

## 1. Introduction

Rheumatoid arthritis (RA) is a chronic inflammatory disease characterized by recruitment and activation of inflammatory cells such as macrophages and helper T cells in the synovia [1].

Macrophages aggravate the inflammatory process in diseases such as RA by releasing cytokines. Tumor necrosis factor-alpha (TNF-*α*), a proinflammatory cytokine predominantly produced by macrophages, is actively involved in the inflammatory process and destruction of joints in RA [2, 3]. Evidence has shown that TNF-*α* exists in the synovial fluid and synovial tissues in patients with RA [4, 5]. TNF-*α* antibodies and soluble TNF-*α* receptors are effective in RA patients and animal models of RA [6–8]. IL-10, in turn, acts as anti-inflammatory cytokine to control the production of TNF-*α* by active macrophages [9, 10].

Helper T cells can be divided into Th1 and Th2 subsets in two ways: the first way is based on their cytokine secretion profile [11]. That is, Th1 cells produce interferon-gamma (IFN-*γ*) and interleukin-2 (IL-2) but neither IL-4 nor IL-5, while Th2 cells produce IL-4 and IL-5 but not IL-2 or IFN-*γ*. The second way is by the expression of receptors for chemokines on the cell surface. The CXC chemokine receptor CXCR3 is expressed in Th1 cells, whereas the CC chemokine receptor CCR4 is expressed in Th2 cells [12–14]. An imbalanced Th1/Th2 response has been suggested to be involved in the progression of RA. That is, a predominance of Th1 cells over Th2 cells aggravates the RA [15].

Adjuvant arthritis (AA), a widely studied model of human RA, is also characterized by the recruitment of macrophages and helper T cells to the synovial tissues [16]. The percentage of both macrophages and helper T cells in the mononuclear cell population in the synovia of ankle joints is consistent with the swelling in the hind paws of AA rats [17]. Studies have suggested that TNF-*α* and IFN-*γ* contribute to the progression, whereas IL-10 and IL-4 contribute to the remission, of AA [18, 19].

Boi-ogi-to (TJ-20), a Kampo formula consisting of six ingredients,* Astragali* radix,* Sinomenium acutum*, Atractylodes Lancea rhizoma,* Zizyphi fructus*,* Glycyrrhizae* radix, and* Zingiber* rhizome, has been used for centuries in China and Japan for the treatment of RA. Although an ameliorating effect of* Sinomenium acutum*, a main ingredient of TJ-20 (Table 1), has been reported in rat experimental arthritis and in the treatment of rheumatic diseases [20, 21], the immunological aspect of the effect of TJ-20 in RA is still unclear.

In this study, we examine the therapeutic effects of TJ-20 on the expression of cytokines and the Th1/Th2 balance in the synovia of AA rats, to obtain scientific proof of the clinical effectiveness of TJ-20.

## 2. Methods

### 2.1. Induction of AA and Measurement of Paw Swelling

Thirty-six female Lewis rats, weighing 100–110 g, were treated in accordance with the guidelines stipulated by the animal care and use committee of Kyushu University. Twenty-four rats received a single intradermal injection of complete Freund's adjuvant (CFA; heat-killed* Mycobacterium butyricum* were well suspended in mineral oil, 10 mg/ml, 25 mg/kg; Difco Lab., Detroit, MI, USA) at the base of tail under deep ether anesthesia. Twelve rats injected with mineral oil only served as controls (normal rats) for the adjuvant injection. The size (thickness of the ankle from the medial to lateral malleolus) of the hind paws was measured from the day after adjuvant injection (day 0) using digimatic micrometer (Mitutoyo, Kanagawa, Japan). The percent of increase was compared with that of day 0.

### 2.2. Administration of TJ-20

TJ-20 bulk powder (Tsumura & Co. Ltd., Tokyo, Japan) was dissolved in distilled water (100 mg/ml). Eighteen rats injected with adjuvant were orally administered TJ-20 (600 mg/kg) once a day according to three different schedules (six rats per schedule): schedule I: from day 0 to week 8; schedule II: from day 7 to week 8; schedule III: from day 10 to week 8. Distilled water was orally administered to a group of six rats as adjuvant arthritic controls (AA rats), while TJ-20 was administered to six rats from day 0 as TJ-20 treatment controls (control rats).

### 2.3. Tissue Preparations

The rats were anesthetized with Nembutal (30 mg/kg) at the end of each experimental schedule, perfused intracardially with 0.01 M phosphate-buffered saline (PBS, pH 7.4), and fixed in PLP fixative (0.01 M sodium metaperiodate; 0.075 M L-lysine-HCl; 2% paraformaldehyde, 0.03 M PB, pH 6.2). Ankle joints, including the tarsal bone and tibia, were excised, immersed in the same fixative for 6 h at 4°C, then washed with PBS, and decalcified in 10% EDTA for 3 weeks at 4°C. After decalcification, specimens were embedded in Optimal Cutting Temperature (OCT) compound (Sakura Finetechnical Co., Ltd., Tokyo, Japan). Serial frozen sections (7 *µ*m) of ankle joints for staining with hematoxylin and eosin and immunohistochemistry were prepared as reported previously [17].

### 2.4. Immunohistochemistry

Sections were treated with 0.3% H_2_O_2_ in methanol and then were treated with 10% normal donkey serum for 2 h at 24°C. The sections were incubated with the mouse anti-W3/25 (1 : 200; Harlan Sera-Lab Lid, Loughborough, England), goat anti-IL-4 (1 : 100; BD PharMingen), goat anti-IFN-*γ* (1 : 100; Santa Cruz Biotechnology), goat anti-ED1 (1 : 500; Santa Cruz Biotechnology), goat anti-TNF-*α* (1 : 100; Santa Cruz Biotechnology), and goat anti-IL-10 (1 : 100; Santa Cruz Biotechnology) antibodies in a humidified chamber overnight at 4°C. After being washed with cold PBS, the sections were incubated with biotinylated-anti-mouse secondary antibodies (1 : 200; Jackson ImmunoResearch) or biotinylated-anti-goat secondary antibodies (1 : 200; Jackson ImmunoResearch) for 2 h at 24°C and finally with peroxidase conjugated streptavidin (1 : 300; Dako) for 1 h at 24°C. The peroxidase was developed using 3,30-diaminobenzidine (DAB substrate kit; Vector Laboratories), and then the samples were counterstained with Mayer's hematoxylin.

### 2.5. Double-Immunofluorescence Staining

The sections were hydrated and treated with 10% normal donkey serum for 1 h at 24°C and then incubated with the following antibodies for 2 days at 4°C: goat anti-CXCR3 (1 : 100; Santa Cruz Biotechnology) with mouse anti-W3/25 (1 : 200; Harlan Sera-Lab Lid, Loughborough, England); goat anti-CCR4 (1 : 100; Santa Cruz Biotechnology); and mouse anti-W3/25 (1 : 200; Harlan Sera-Lab Lid, Loughborough, England). The sections were washed with PBS and incubated with a mixture of secondary antibodies conjugated with donkey anti-goat Alexa 488 (1 : 400; Jackson ImmunoResearch) and donkey anti-mouse Cy3 (1 : 400; Jackson ImmunoResearch) for 3 h at 24°C. The sections were washed with PBS and mounted in the antifading medium Vectashield (Vector Laboratories) and examined with a confocal laser-scanning microscope (CLSM) (C2si Confocal Laser Microscope, Nikon).

### 2.6. Statistical Analysis

The percent increase in hind paw swelling and body weight gain of the rats on each schedule were calculated and expressed as the mean ± SD. Mononuclear cells were counted under a light microscope using the 40x objective and 10x ocular lenses in three areas per section (3 sections per rat). The percentages of ED-positive cells or W3/25-positive cells in mononuclear cells in each area were calculated (the percentage of the positive mononuclear cells) and expressed as the mean ± SD. The data are presented as the means ± SEM. The statistical analyses of the results were performed with Student's unpaired *t*-tests and one-way ANOVA with a post hoc Tukey's tests using the GraphPad Prism software package. A value of *P* < 0.05 was considered to indicate statistical significance.

## 3. Results

### 3.1. Oral Administration of TJ-20 Suppresses the Inflammatory Progression and Bone Destruction of Adjuvant Arthritis

**Figure 1:**
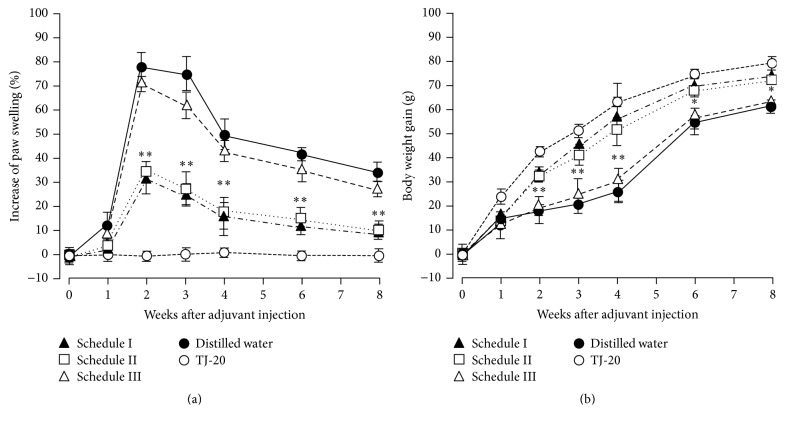
Time course of the suppressive effects of TJ-20 on the clinical parameters of AA rats. AA rats were given TJ-20 according to schedule I (filled triangles), II (open squares), or III (open triangles) or given distilled water (closed circle) orally once a day after adjuvant injection (AA rats). Rats injected with mineral oil alone and given TJ-20 (open circle) served as controls. The hind paw swelling (a) and body weight gain (b) were measured at 1-week intervals. TJ-20 administered according to schedules I and II significantly ameliorated the progression of AA. Data are the mean ± SD for 6 rats in each group. Asterisks indicate a statistically significant difference from AA rats (^*∗*^*P* < 0.05, ^*∗∗*^*P* < 0.01).

The clinical symptoms, such as redness and swelling of the hind paws in the AA rats, appeared on day 10, peaked at week 2, and remained until week 3. Although the redness and swelling declined thereafter, the hind paws did not recover completely until week 8 (Figure 1(a)). AA rats lost body weight from day 10 to week 3 but gained weight gradually thereafter (Figure 1(b)). The normal rats did not show any clinical symptoms and had gained body weight gradually by week 6 (data not shown). Compared with AA rats, significant reductions in the hind paw swelling and body weight loss were detected at weeks 2 to 3 in the rats given TJ-20 according to schedules I and II, and no significant differences in clinical symptoms were found between these two groups. However, no significant amelioration of swelling and weight loss was detected in the AA rats on schedule III. Control rats did not show any clinical symptoms and gained body weight following a similar time course as the normal rats (Figures 1(a) and 1(b)).

**Figure 2:**
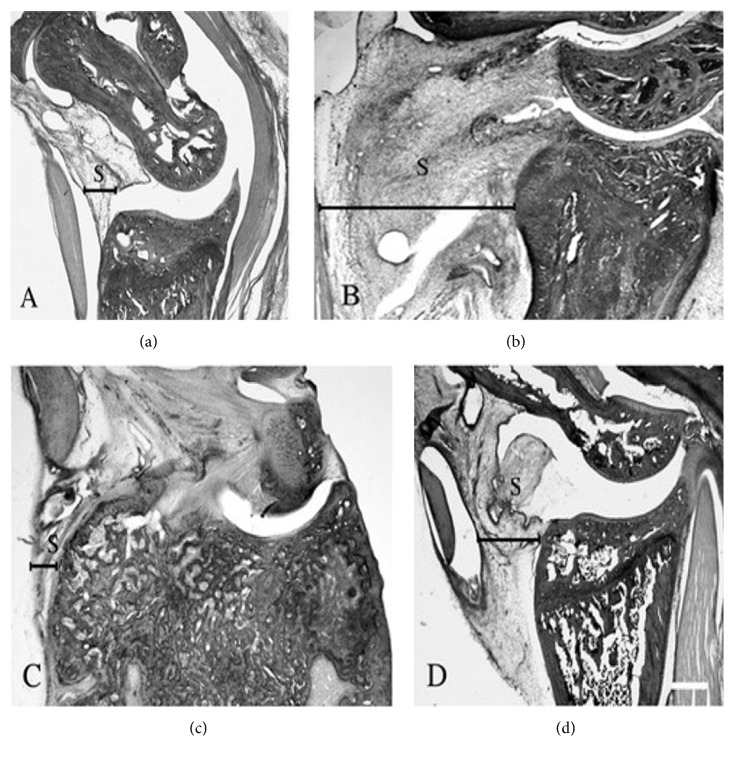
Histological examination of the ankle joints in AA rats. Hematoxylin-eosin staining in ankle joints from control rats (a) and AA rats at week 2 with a remarkable inflammation in synovia (b) and week 8 with the remarkably destruction of cartilage and bone (c) after adjuvant injection. The thickness of the synovia was markedly suppressed in AA rats treated with TJ-20 according to schedule II (d) at week 2 after adjuvant injection. S: synovia; scale bar, 200 *µ*m.

In the histological examination, compared with control rats (Figure 2(a)), a significant recruitment of mononuclear cells was observed at day 7 and the thickness of the synovia reached a maximal level at weeks 2 to 3 (Figure 2(b)). Progress in the destruction of cartilage and bone from weeks 3 to 8 was apparent in AA rats with a decrease of inflammation in synovia (Figure 2(c)). Among the AA rats, we detected a marked suppression of the thickening of the synovia (Figure 2(d)) and of the destruction of bone in the rats treated with TJ-20 according to schedules II and I (data not shown). We did not observe any effects in the AA rats on schedule III (data not shown). These results showed that administration of TJ-20 according to schedules I and II suppressed the progression of arthritis in AA rats.

### 3.2. Influence of TJ-20 on the Recruitment of Macrophages and Helper T Cells in the Synovia of AA Rats

**Figure 3:**
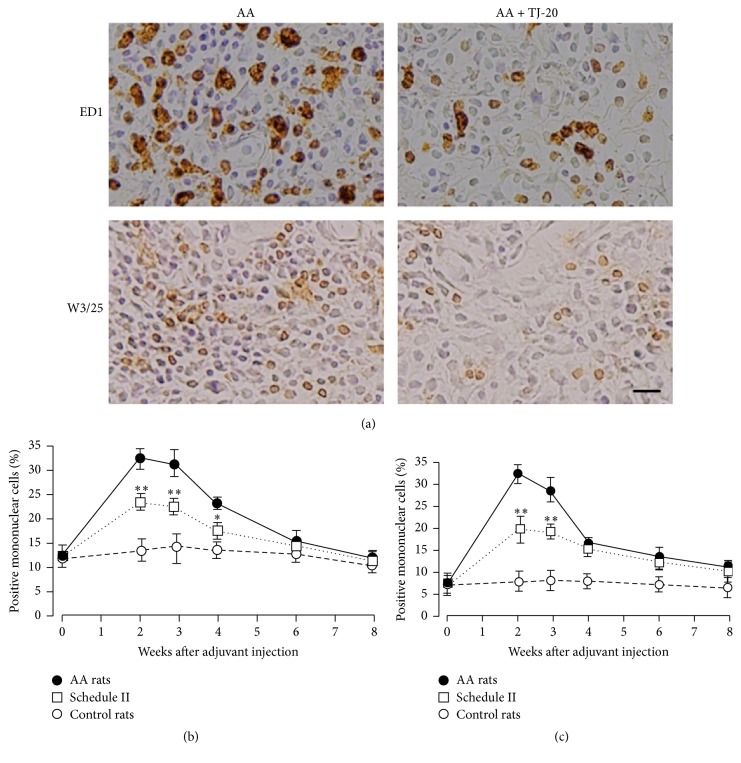
Influence of TJ-20 on the recruitment of macrophages and helper T cells in the synovia of ankle joint in AA rats. Photographs representing ED1-positive macrophages and W3/25-positive helper T cells in the synovia of AA rats at week 2 and AA rats treated with TJ-20 according to schedule II (a), the percentages of ED1-positive macrophages (b), and W3/25-positive helper T cells (c) in AA rats (closed circle), in AA rats treated with TJ-20 according to schedule II (open squares), and in control rats (open circle) were determined by immunohistochemistry. TJ-20 administered according to schedule II significantly lowered the increase in a number of both macrophages and helper T cells. Data are the mean ± SD for 6 rats in each group. Asterisks indicate a statistically significant difference from AA rats (^*∗*^*P* < 0.05, ^*∗∗*^*P* < 0.01). Scale bar, 25 *µ*m.

To investigate whether TJ-20 treatment influences the recruitment of macrophages and helper T cells in the synovia of AA rats, immunohistochemical staining was carried out using anti-macrophage antibodies (ED1) and anti-T cell antibodies (W3/25). In the synovia of AA rats, we observed a similar time course of change in the percentage of ED1-positive cells and W3/25-positive cells in total mononuclear cells. These cell populations increased significantly from day 10 and reached a maximal level during weeks 2 and 3 (Figures 3(a)–3(c)) and then decreased from week 4 after the adjuvant was injected. Significant reductions in the percentage of ED1-positive mononuclear cells from weeks 2 to 4 (Figures 3(a) and 3(b)) and W3/25-positive mononuclear cells from weeks 2 to 3 (Figures 3(a) and 3(c)) were detected in the synovia of AA rats treated with TJ-20 according to schedules II and I (data not shown). However, no significant decrease in the percentage of ED1-positive or W3/25-positive mononuclear cells was observed in the synovia of AA rats treated with TJ-20 using schedule III (data not shown). These results showed that administration of TJ-20 according to schedules I and II suppressed the infiltration of both macrophages and helper T cells into the synovia of AA rats until week 4 after injection of the adjuvant.

### 3.3. Influence of TJ-20 on the Expression of Cytokines in the Synovia of AA Rats

**Figure 4:**
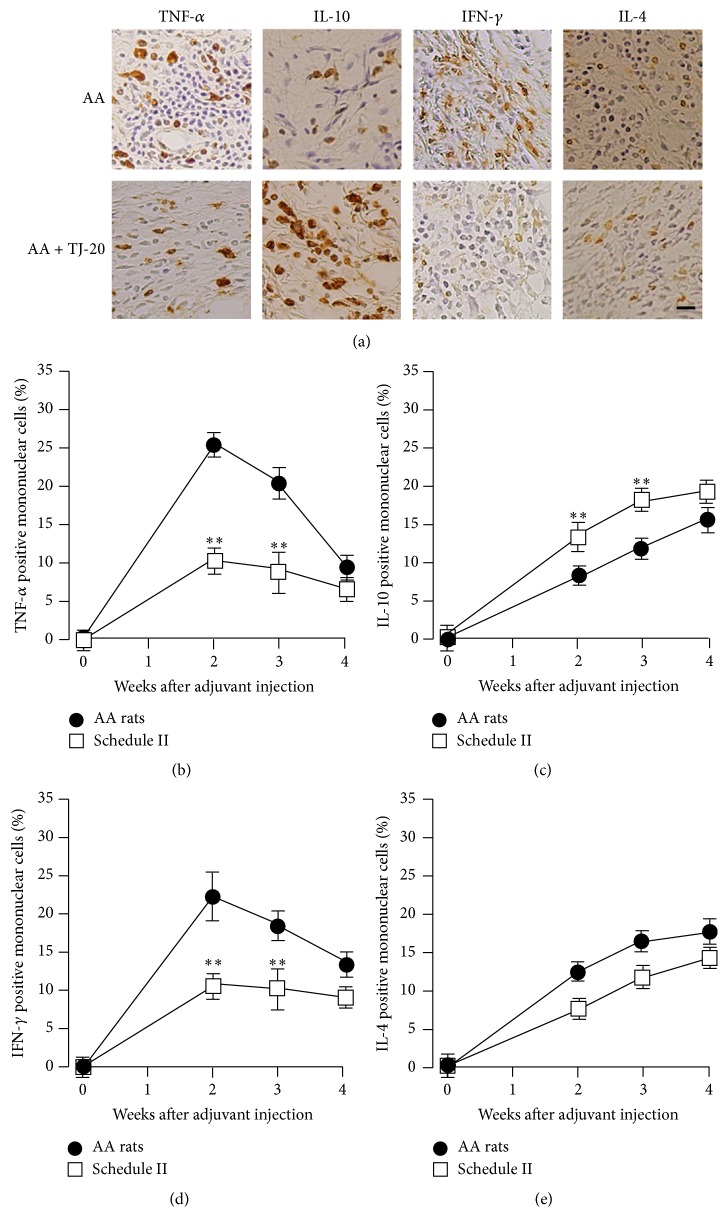
Influence of TJ-20 on the expression of cytokines in the synovia of ankle joint in AA rats. Photographs representing TNF-*α*-, IL-10-, IFN-*γ*-, and IL-4-positive mononuclear cells in the synovia of AA rats at week 2 and AA rats treated with TJ-20 according to schedule II (a), the percentages of TNF-*α*-positive (b), and IL-10-positive (c), IFN-*γ*-positive (d), and IL-4-positive (e) mononuclear cells in AA rats (closed circle) and AA rats treated with TJ-20 according to schedule II (open squares) were determined by immunohistochemistry. TJ-20 administered according to schedule II significantly suppressed the expression of TNF-*α* and IFN-*γ* and augmented the expression of IL-10 but did not augment the expression of IL-4. Asterisks indicate a statistically significant difference from AA rats (^*∗*^*P* < 0.05, ^*∗∗*^*P* < 0.01). Scale bar, 25 *µ*m.

To investigate whether TJ-20 treatment influences the expression of proinflammatory cytokines and anti-inflammatory cytokines in the synovia in AA rats, we examined the percentage of TNF-*α*-and IL-10-positive mononuclear cells in the ankle joints. TNF-*α*-positive cells were detected from day 7. The percentage of TNF-*α*-positive cells (Figure 4(a)) in total mononuclear cells increased significantly and reached their maximum at week 2 and then decreased significantly from weeks 3 to 4 (Figure 4(b)). IL-10-positive cells (Figure 4(a)) were also detected from day 7, but the percentage increased gradually from weeks 2 to 4 after injection of adjuvant (Figure 4(c)). In comparison with AA rats, we observed a significant decrease in the percentage of TNF-*α*-positive mononuclear cells (Figures 4(a) and 4(b)) and in contrast, a significant increase in the percentage of IL-10-positive mononuclear cells from weeks 2 to 4 in the synovia of AA rats treated with TJ-20 according to schedules II (Figures 4(a) and 4(c)) and I (data not shown). No significant change in the percentage of TNF-*α*- or IL-10-positive mononuclear cells was observed in the AA rats treated with TJ-20 using schedule III (data not shown). These results showed that administration of TJ-20 according to schedules I and II suppressed the recruitment of TNF-*α*-expressing cells but increased that of IL-10-expressing cells in the synovia of AA rats.

### 3.4. Effects of TJ-20 on Th1/Th2 Balance in the Synovia of AA Rats

To investigate whether TJ-20 treatment influences the balance of Th1/Th2 cells in the synovia in AA rats, we examined the percentages of IFN-*γ*-positive (one of the Th1 cytokines, Figure 4(a)) and IL-4-positive (one of the Th2 cytokines, Figure 4(a)) mononuclear cells and also the percentages of CXCR3 (a marker for Th1 cells)/W3/25-double and CCR4 (a marker for Th2 cells)/W3/25-double positive cells in the synovia of the ankle joints (data not shown). IFN-*γ*-positive cells were detected on day 7. The percentage among mononuclear cells was increased markedly and reached its maximum at week 2 and then decreased significantly from week 3 (Figure 4(d)). In contrast, the percentage of IL-4-positive mononuclear cells increased significantly from weeks 2 to 4 after the injection of adjuvant (Figure 4(e)). The percentage of CXCR3/W3/25-double positive cells also increased markedly at week 2 but decreased significantly at week 4, while the percentage of CCR4/W3/25-positive cells increased significantly from weeks 2 to 4. The ratio of CXCR3/W3/25- to CCR4/W3/25-double positive cells changed from 2.22 at week 2 to 0.75 at week 4 after injection of the adjuvant (Table 2). In comparison with AA rats, we have detected a significant decrease in both the percentages of IFN-*γ*-positive mononuclear cells (Figure 4(d)) and the percentage of CXCR3/W3/25-double positive cells from weeks 2 to 3 (Table 2); however we could not detect any significant difference in either the percentage of IL-4-positive mononuclear cells (Figure 4(e)) or the percentage of CCR4/W3/25-double positive cells detected from weeks 2 to 4 in the synovia of AA rats treated with TJ-20 according to schedules II and I (data not shown). The ratio of CXCR3/W3/25- to CCR4/W3/25-double positive cells changed from 1.57 in week 2 to 0.62 in week 4 after injection of the adjuvant (Table 2). These results strongly suggest that the administration of TJ-20 using schedules I and II significantly suppressed the recruitment of Th1 cells into the synovia of AA rats.

## 4. Discussion

Drugs are metabolized in animals more quickly than in humans [22]. It was reported that the hepatic clearance of drugs in humans was approximately one-seventh of that in other mammals including rats [23]. Furthermore, it is known that the renal clearance rate of mice is about tenfold greater than that of humans [24]. Therefore, in the present study, TJ-20 was administered to rats at a dose of 600 mg per kg of body weight daily, which is equivalent to tenfold the clinical dosage used in humans. Since no body weight loss was observed in the control rats and no significant difference in the body weight gain was detected between the AA rats given TJ-20 on schedule I or II and the normal rats, it is suggested that TJ-20 has few side effects.

Adjuvant arthritis in rats is often used for the assessment of antirheumatic drugs [20, 21, 25]. After injection of adjuvant, mononuclear cells were detected in the synovia as induction phages on day 4, and the swelling of the joints was observed on day 10, as the signs of the onset of AA [26]. From weeks 2 to 3, marked infiltration by mononuclear cells is apparent, represented as the acute phases. After week 4, a significant decrease in the number of mononuclear cells in the synovia was observed, corresponding to the chronic phases of AA [17, 26]. It is suggested that the activation of macrophages and helper T cells as well as the increase in the production of mediators is essential for the onset of AA [27]. In the present study, we found that administration of TJ-20 from day 0 or from day 7 (schedule I and II), but not from day 10 (schedule III), significantly suppressed the progress of AA. Therefore, we hypothesized that TJ-20 affects the infiltration of macrophages and helper T cells into the synovia during the induction stage of AA, but cannot adequately suppress the function of activated macrophages and helper T cells after the onset of AA.

Macrophages and T lymphocytes are considered pivotal to the pathogenesis of RA [1]. Previously, we have reported that the time course of changes in the percentage of both macrophages and helper T cells among mononuclear cells in the synovia is consistent with the swelling in the hind paws of AA rats [17]. Suppression of the activation of both macrophages and T lymphocyte seems as a more effective treatment for RA [28]. Common antirheumatic drugs for RA, such as methotrexate, can inhibit activation of T lymphocytes [29] and macrophages [30]. In this study, we found that TJ-20 administration from induction phases significantly suppressed the percentages of both ED1-positive cells and W3/25-positive cells among mononuclear cells in the synovia of ankle joints of AA rats. These results suggest that TJ-20 can reduce the infiltration of macrophages and T cells into the synovia in AA rats.

The overproduction of TNF-*α* and IL-10 in the synovia in RA is reported [31]. It has also been reported that the increased level of TNF-*α* in the synovial fluid and synovia is associated with joint destruction in RA [4, 5]. On the other hand, IL-10 suppresses the production of TNF-*α* [9, 10] and progression of arthritis [32, 33]. In AA rats, it has been reported that TNF-*α* is involved in the progression while IL-10 is involved in the remission of AA [18, 19]. In the present study, a significant decrease in the percentage of TNF-*α*-positive cells and a significant increase of the percentage of IL-10-positive cells were detected in AA rats treated with TJ-20 from the induction phases of AA. Since the suppression of TNF-*α* production and augmentation of IL-10 expression are thought to be a favorable approach for treating RA [34–36], we speculated that the administration of TJ-20 could be an ideal therapy for arthritis.

An imbalance of Th1/Th2 is also related the progression of RA [37]. IFN-*γ*, one of the Th1 cytokines, enhanced the aggravation of RA [15, 38]. IL-4, one of the Th2 cytokines, suppressed the inflammation and joint destruction of RA [39]. In AA rats, IFN-*γ* is shown to be involved in the progression of AA, while IL-4 is involved in the remission of AA, respectively [18, 19]. Chemokines receptors have been reported as markers for Th1 and Th2 cells [12–14]. Using these markers, we have shown that the increase in the number of Th1 cells is highly associated with the progression of AA, while the increase in the number of Th2 cells is involved in its remission [15]. The current study showed that administration of TJ-20 from the induction phases significantly reduced the recruitment of Th1 cells, suggesting that mainly Th1 cells are influenced by TJ-20. Th2 cells have a suppressive effect on Th1 cells through the production of cytokines [40, 41]; however, administration of TJ-20 did not influence either the number of IL-4-expressing cells or that of CCR4-positive helper T cells in the synovia of AA rats. Our observation suggests that the TJ-20 treatment did not affect the recruitment of Th2 cells. Therefore, we speculate that the therapeutic effects of TJ-20 on AA involve regulation of the Th1/Th2 balance. The anti-inflammatory effects of* Sinomenium acutum* and Atractylodes Lancea rhizome, the main ingredients of TJ-20, may contribute to the effects of TJ-20 on AA rats [20, 42].

**Figure 5:**
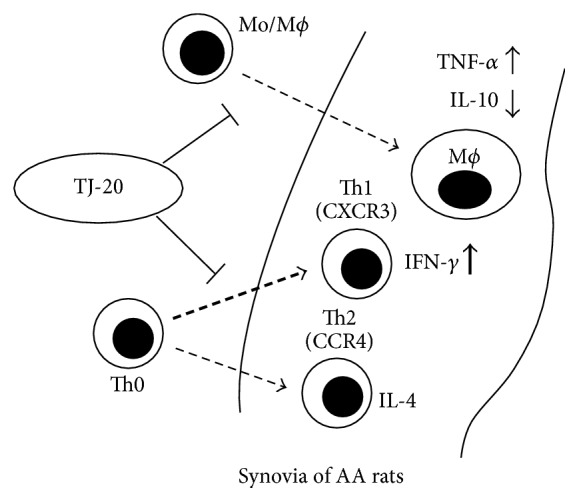
A schematic representation of the effects and the principle molecular mechanisms of TJ-20 on adjuvant arthritis. TJ-20 reduces the recruitment of macrophages and helper T cells into the synovia of ankle joint in AA rats. Consequently, TJ-20 downregulates the production of TNF-*α* but augments the production of IL-10, which are mainly released by macrophages; TJ-20 also regulates the balance of Th1/Th2 ratio as it suppresses a recruitment of Th1 cells in the synovia of AA rats.

In conclusion, the current study demonstrated that oral administration of TJ-20 during the induction phase of AA ameliorated the progression of AA in rats. TJ-20 might suppress the recruitment of inflammatory cells, both macrophages and helper T cells, into the synovia. Such pharmacological events could regulate the expression of proinflammatory cytokines/anti-inflammatory cytokines, leading to a modulation of the Th1/Th2 balance in the synovia in AA rats (Figure 5). These results provide a scientific basis for the clinical use of TJ-20 for RA and indicate that TJ-20 is useful for treating at early stages of RA. Further studies are required concerning the effects of TJ-20 on the destruction of bone in the arthritic joints.

## Figures and Tables

**Table 1:** Composition of Boi-ogi-to (TJ-20).

Medicinal plants	Weight ratio	Family	Genus	Species
Astragali radix	5	Leguminosae	Astragalus	Astragalus
*Sinomenium acutum*	5	Menispermaceae	Sinomenium	Sinomenium acutum
Atractylodis Lanceae rhizoma	3	Asteraceae	Atractylodes	Lancea
Jujube fruit	3	Rhamnaceae	Ziziphus	Zizyphus
Glycyrrhizae radix	1.5	Leguminosae	Glycyrrhiza	Glycyrrhiza
Zingiberis Rhizoma	1	Zingiberaceae	Zingiber	Zingiberis Rhizoma

**Table 2:** Effects of TJ-20 on Thl/Th2 balance in the synovia of ankle joint in AA rats.

Time	AA rats			AA rats treated with TJ-20		
	CXCR3 (Th1)	CCR4 (Th2)	CXCR3/CCR4 Th1/Th2 ratio	CXCR3 (Th1)	CCR4 (Th2)	CXCR3/CCR4 Th1/Th2 ratio
2 weeks	60 ± 6.5	27 ± 4.3	2.22	46.9 ± 5.2^*∗∗*^	29.8 ± 4.2	1.57
3 weeks	52 ± 5.7	44 ± 7.2	1.18	43.1 ± 6.1^*∗*^	44.6 ± 3.8	0.97
4 weeks	42 ± 4.8	56 ± 6.7	0.75	38 ± 3.6	61.6 ± 3.1	0.62

By use of doubled-immunofluorescence study, we have determined the percentages of CXCR3/W3/25-double-positive cells and CCR4/W3/25-double-positive cells among total W3/25-positive cells in AA rats and AA rats with TJ-20 administration from day 7 after adjuvant injection. Data are the mean ± SD for 6 rats in each group. Asterisks indicate a statistically significant difference from AA rats (^*∗*^*P* < 0.05, ^*∗∗*^*P* < 0.01).

## References

[1] Janossy G., Panayi G., Duke O., Bofill M., Poulter L. W., Goldstein G. (1981). Rheumatoid arthritis: a disease of T-lymphocyte/macrophage immunoregulation. *The Lancet*.

[2] McInnes I. B., Schett G. (2007). Cytokines in the pathogenesis of rheumatoid arthritis. *Nature Reviews Immunology*.

[3] Netea M. G., Radstake T., Joosten L. A. (2003). Salmonella septicemia in rheumatoid arthritis patients receiving anti-tumor necrosis factor therapy: association with decreased interferon-gamma production and Toll-like receptor 4 expression. *Arthritis and Rheumatism*.

[4] Genovese M. C., Becker J.-C., Schiff M. (2005). Abatacept for rheumatoid arthritis refractory to tumor necrosis factor *α* inhibition. *The New England Journal of Medicine*.

[5] Marotte H., Maslinski W., Miossec P. (2005). Circulating tumour necrosis factor-*α* bioactivity in rheumatoid arthritis patients treated with infliximab: link to clinical response. *Arthritis Research & Therapy*.

[6] Maini R. N., Elliott M. J., Brennan F. M. (1995). Monoclonal anti-TNF alpha antibody as a probe of pathogenesis and therapy of rheumatoid disease. *Immunological Reviews*.

[7] Bush K. A., Walker J. S., Frazier J., Kirkham B. W. (2002). Effects of a PEGylated soluble TNF receptor type 1 (PEG sTNF-RI) on cytokine expression in adjuvant arthritis. *Scandinavian Journal of Rheumatology*.

[8] Brezinschek H.-P., Hofstaetter T., Leeb B. F., Haindl P., Graninger W. B. (2008). Immunization of patients with rheumatoid arthritis with antitumor necrosis factor*α* therapy and methotrexate. *Current Opinion in Rheumatology*.

[9] Fiorentino D. F., Zlotnik A., Mosmann T. R., Howard M., O'Garra A. (1991). IL-10 inhibits cytokine production by activated macrophages. *Journal of Immunology*.

[10] Trindade M. C. D., Lind M., Nakashima Y. (2001). Interleukin-10 inhibits polymethylmethacrylate particle induced interleukin-6 and tumor necrosis factor-*α* release by human monocyte/macrophages in vitro. *Biomaterials*.

[11] Mosmann T. R., Coffman R. L. (1989). Heterogeneity of cytokine secretion patterns and functions of helper T cells. *Advances in Immunology*.

[12] Yamamoto J., Adachi Y., Onoue Y. (2000). Differential expression of the chemokine receptors by the Th1- and Th2-type effector populations within circulating CD4+ T cells. *Journal of Leukocyte Biology*.

[13] Langenkamp A., Nagata K., Murphy K., Wu L., Lanzavecchia A., Sallusto F. (2003). Kinetics and expression patterns of chemokine receptors in human CD4+ T lymphocytes primed by myeloid or plasmacytoid dendritic cells. *European Journal of Immunology*.

[14] Wu Z., Toh K., Nagata K., Kukita T., Iijima T. (2004). Effect of the resection of the sciatic nerve on the Th1/Th2 balance in the synovia of the ankle joint of adjuvant arthritic rats. *Histochemistry and Cell Biology*.

[15] Dolhain R. J., van der Heiden A. N., ter Haar N. T., Breedveld F. C., Miltenburg A. M. (1996). Shift toward T lymphocytes with a T helper 1 cytokine-secretion profile in the joints of patients with rheumatoid arthritis. *Arthritis & Rheumatology*.

[16] Pelegri C., Franch A., Castellote C., Castell M. (1995). Immunohistochemical changes in synovial tissue during the course of adjuvant arthritis. *Journal of Rheumatology*.

[17] Wu Z., Nagata K., Iijima T. (2000). Immunohistochemical study of NGF and its receptors in the synovial membrane of the ankle joint of adjuvant-induced arthritic rats. *Histochemistry and Cell Biology*.

[18] Schmidt-Weber C. B., Pohlers D., Siegling A. (1999). Cytokine gene activation in synovial membrane, regional lymph nodes, and spleen during the course of rat adjuvant arthritis. *Cellular Immunology*.

[19] Hossain A., Zheng C. L., Kukita A., Kohashi O. (2001). Balance of Th1/Th2 cytokines associated with the preventive effect of incomplete Freund's adjuvant on the development of adjuvant arthritis in LEW rats. *Journal of Autoimmunity*.

[20] Yamasaki H. (1976). Pharmacology of sinomenine, an anti-rheumatic alkaloid from Sinomenium acutum. *Acta Medica Okayama*.

[21] Liu L., Buchner E., Beitze D. (1996). Amelioration of rat experimental arthritides by treatment with the alkaloid sinomenine. *International Journal of Immunopharmacology*.

[22] Brodie B. B. (1964). Difficulties in transposing experimental results obtained with animals to man. *Actualites pharmacologiques*.

[23] Boxenbaum H. (1980). Interspecies variation in liver weight, hepatic blood flow, and antipyrine intrinsic clearance: extrapolation of data to benzodiazepines and phenytoin. *Journal of Pharmacokinetics and Biopharmaceutics*.

[24] Dedrick R., Bischoff K. B., Zaharko D. S. (1970). Interspecies correlation of plasma concentration history of methotrexate (NSC-740). *Cancer Chemother Reports*.

[25] Zhang T., Li H., Shi J. (2016). p53 predominantly regulates IL-6 production and suppresses synovial inflammation in fibroblast-like synoviocytes and adjuvant-induced arthritis. *Arthritis Research & Therapy*.

[26] Pearson C. M., Wood F. D. (1963). Studies of arthritis and other lesions induced in rats by the injection of mycobacterial adjuvant. VII. Pathologic details of the arthritis and spondylitis. *The American Journal of Pathology*.

[27] Taurog J. D., Sandberg G. P., Mahowald M. L. (1983). The cellular basis of adjuvant arthritis. II. Characterization of the cells mediating passive transfer. *Cellular Immunology*.

[28] FitzGerald O. (1995). Advances in understanding and novel therapeutic targets in inflammatory arthritis. *Irish Journal of Medical Science*.

[29] Neurath M. F., Hildner K., Becker C. (1999). Methotrexate specifically modulates cytokine production by T cells and macrophages in murine collagen-induced arthritis (CIA): a mechanism for methotrexate-mediated immunosuppression. *Clinical and Experimental Immunology*.

[30] Smith M. D., Kraan M. C., Slavotinek J. (2001). Treatment-induced remission in rheumatoid arthritis patients is characterized by a reduction in macrophage content of synovial biopsies. *Rheumatology*.

[31] Klareskog L., Rönnelid J., Holm G. (1995). Immunopathogenesis and immunotherapy in rheumatoid arthritis: an area in transition. *Journal of Internal Medicine*.

[32] Walmsley M., Katsikis P. D., Abney E. (1996). Interleukin-10 inhibition of the progression of established collagen-induced arthritis. *Arthritis & Rheumatology*.

[33] Persson S., Mikulowska A., Narula S., O'Garra A., Holmdahl R. (1996). Interleukin-10 suppresses the development of collagen type II-induced arthritis and ameliorates sustained arthritis in rats. *Scandinavian Journal of Immunology*.

[34] Hwang D., Kim W. (2017). Rheumatoid arthritis: modelling cytokine signaling networks. *Nature Reviews Rheumatology*.

[35] Szekanecz Z., Koch A. E., Kunkel S. L., Strieter R. M. (1998). Cytokines in rheumatoid arthritis. Potential targets for pharmacological intervention. *Drugs and Aging*.

[36] Hisadome M., Fukuda T., Sumichika H., Hanano T., Adachi K. (2000). A novel anti-rheumatic drug suppresses tumor necrosis factor-*α* and augments interleukin-10 in adjuvant arthritic rats. *European Journal of Pharmacology*.

[37] Schulze-Koops H., Kalden J. R. (2001). The balance of Th1/Th2 cytokines in rheumatoid arthritis. *Best Practice and Research: Clinical Rheumatology*.

[38] Gerli R., Bistoni O., Russano A. (2002). In vivo activated T cells in rheumatoid synovitis. Analysis of Th1- and Th2-type cytokine production at clonal level in different stages of disease. *Clinical and Experimental Immunology*.

[39] van Roon J. A., Lafeber F. P., Bijlsma J. W. (2001). Synergistic activity of interleukin-4 and interleukin-10 in suppression of inflammation and joint destruction in rheumatoid arthritis. *Arthritis & Rheumatology*.

[40] Zhu J., Yamane H., Paul W. E. (2010). Differentiation of effector CD4+ T cell populations. *Annual Review of Immunology*.

[41] Peine M., Rausch S., Helmstetter C. (2013). Stable T-bet^+^GATA-3^+^ Th1/Th2 hybrid cells arise in vivo, can develop directly from naive precursors, and limit immunopathologic inflammation. *PLoS Biology*.

[42] Atsumi T., Iwashita A., Ohtsuka I. (2013). Effects of atractylodis lanceae rhizoma on inflammatory mediator production from the RAW264 macrophage cell line. *Journal of Traditional Medicines*.

